# Cementless unicompartmental knee arthroplasty results in higher pain levels compared to the cemented technique: a prospective register study

**DOI:** 10.1007/s00167-021-06617-5

**Published:** 2021-05-25

**Authors:** Tone Gifstad, Jørgen Jebens Nordskar, Tarjei Egeberg, Tina Strømdal Wik, Siri Bjørgen Winther

**Affiliations:** 1 Department of Orthopaedics, Trondheim University Hospital, Postbox 3250, NO 7006 Torgarden, Trondheim Norway; 2 Faculty of Medicine, Norwegian University of Science and Technology, Trondheim, Norway; 3 Department of Orthopaedics, Orthopaedic Research Center, Trondheim University Hospital, Trondheim, Norway

**Keywords:** Unicompartmental knee arthroplasty, PROMs, Registry, Cemented, Cementless, Fixation technique

## Abstract

**Purpose:**

In recent years, the preferred fixation method for unicompartmental knee arthroplasty (UKA) has changed from cemented to cementless. The aim of this study was to compare patient-reported outcome measures (PROMs) from the cemented versus cementless techniques two- and twelve-months post-operation.

**Methods:**

From 2015 to 2019, 187 cemented and 261 cementless UKAs were included based on an institutional registry. The Oxford Unicompartmental Knee System™ (Zimmer Biomet, Bridgend, United Kingdom) was used for all patients. Three experienced surgeons performed all procedures. Data were collected pre- and peroperatively, and at two- and twelve-months postoperatively. PROMs included pain (evaluated on a numeric rating scale [NRS] during activity and at rest), and knee function (evaluated with the disease-specific short form of the Knee injury and Osteoarthritis Outcome Score [KOOS-PS]). Patients also rated postoperative joint function (better, unchanged, uncertain or worse) and were asked, “based on your experience to date, would you go through the surgery again?”. Duration of surgery was noted and revisions during the first post-operative year were evaluated.

**Results:**

The cemented group reported significantly lower activity-related pain at both two- and twelve-month follow-up. This was also the case for pain at rest at twelve-month follow-up, and KOOS-PS at two-month follow-up. Duration of surgery (adjusted for surgeon differences) was eight minutes less on average with the cementless technique. Eleven prosthetic joint infections (PJIs) were found following the cementless fixation technique compared to three using the cemented implant.

**Conclusion:**

UKA cases with cemented implants had lower pain scores during activity two and twelve months after surgery compared with those who had cementless implants. Differences in favor of the cemented group were also found for pain at rest one year after surgery and for KOOS-PS two months after. Surgery was significantly shorter in duration in the cementless group, but a relatively high number of PJIs were found in that same group.

**Level of evidence:**

Level II.

## Introduction

There is an ongoing discussion concerning fixation method in medial unicompartmental knee arthroplasty (UKA). In its annual report for 2020, the Norwegian Arthroplasty Register noted an increasing use of cementless UKA, from 1% in 2014 to nearly 40% in 2019 [1]. Campi et al. [9] concluded in their systematic review that the cementless technique was a safe and effective method. They found that in studies examining this technique, patient-reported outcome measure scores (PROMs), failures, and reoperation and survival rates were similar to those found with the cemented technique [9]. This conclusion was also reflected in other recent findings. For example, Mohammad et al. [14] found lower long-term revision rates for cementless UKA compared with cemented UKA and positive results for cementless UKA for all age groups [15]. Basso and colleagues [5] reported that using a cementless fixation could possibly improve survival rate by reducing problems associated with the cement, such as inappropriate penetration and loose fragments. Other researchers have reported shorter surgery duration with cementless implants [2, 5, 18], and reduction of radiological radiolucent lines, possibly improving implant survival [3]. Cementless fixation being seemingly a safe method with acceptable results regarding implant survival and knee function, was introduced at the present hospital in 2017. However, no study had compared postoperative pain following cemented and cementless UKA. For patients with osteoarthritis, pain and its impact on activity of daily living is the main indication for surgery. Consequently, relief of pain after surgery is an important criteria of success and highly relevant to evaluate in new surgical methods. Therefore, the aim of the present study was to compare patient reported activity-related pain and pain at rest, two and twelve months after cemented versus cementless UKA. Scores on the short form of the Knee Injury and Osteoarthritis Outcome Score (KOOS-PS) were also evaluated, together with duration of surgery and revisions reported during the first postoperative year. The hypothesis was that there would be no significant differences in the above-mentioned PROMs between cemented and cementless UKA.

## Methods

**Fig. 1:**
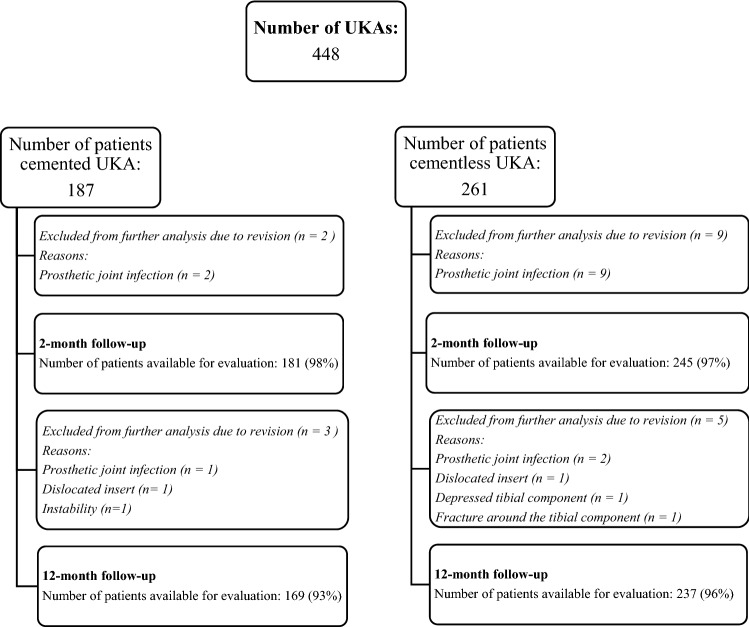
Flow-chart (UKA—unicompartmental knee arthroplasty)

This prospective cohort study was based on an institutional quality registry and included patients undergoing medial UKA between 2015 and 2019. One hundred and eighty-seven patients undergoing surgery from 2015 to 2017 received a cemented UKA, while 261 patients undergoing surgery from 2017 to 2019 received a cementless UKA. A higher number of patients were included in the cementless group in case of a potential learning curve with the new technique. The patients were evaluated preoperatively, and at two- and twelve-month follow-up, by an experienced physiotherapist. The number of patients available at each follow-up is presented in Fig. 1. Three experienced orthopedic surgeons performed all arthroplasties. Preoperatively, patient demographics were collected together with PROMs, presented in Table 1. No significant differences were found in these between the groups. PROMs included pain, reported on a numeric rating scale (NRS) from zero (no pain) to ten during activity and at rest, and KOOS-PS, reported from no difficulty (0) to extreme difficulty (100). At twelve-month follow-up, patients also rated their postoperative joint function (better, unchanged, uncertain or worse) compared with preoperative function and were asked; “based on your experience to date, would you have gone through the surgery again?”. Duration of surgery was also compared for the two groups together with evaluation of postoperative prosthetic joint infection (PJI) and other revisions that occurred during the first year following the operation. All surgeries were carried out following a fast-track model for arthroplasties, and The Oxford Unicompartmental Knee System™ (Zimmer Biomet, Bridgend, United Kingdom) was used during the whole period. The standard technique included a mini-invasive approach through the anteromedial capsule, and microplasty instruments were used. The postoperative treatment was standardized for all patients and focused on immediate weight bearing, early mobilization, multimodal medication for pain relief, thromboembolic prophylaxis and referral to a local physiotherapist [21].

**Table 1:** Mean preoperative data (standard deviation)

	Cemented group	Cementless group
Sex (female/male)	86/101	106/155
Age	64 (9.6)	64 (9.5)
ASA	2 (0.6)	2 (0.6)
BMI (kg/m^2^)	29 (4.9)	30 (4.8)
Pain at activity	6.3 (1.7)	6.4 (1.8)
Pain at rest	3.6 (2.5)	3.6 (2.5)
KOOS-PS	39 (14)	42 (14)

Preoperatively, all patients were informed about the institutional registry for hip- and knee arthroplasty patients and provided written consent to their data being used for scientific purposes. The Regional Committee for Medical and Health Research Ethics, Section A, South East Norway evaluated the project protocol and decided it could be implemented without further approval (IRB00001871 REC South-East A).

### Statistical analysis

A General Linear Mixed Model (GLMM) was used to analyze PROMs and duration of surgery. Age, gender and ASA were included as covariates in all models. All patients undergoing UKA between 2015 and 2019 were included and determined the sample size. The preoperative value of the tested variable was included as a covariate to correct for initial imbalance between the groups. The statistical model used fixation and two time points as fixed factors and included a random subject intercept. Interaction terms were used to obtain group comparisons at two time points. When analyzing duration of surgery, surgeon was included as a fixed factor to account for differences in surgeon-volumes and individual surgeons’ operating-times. Sequential Bonferroni-correction was used to adjust for multiple comparisons. Normality of residuals was verified by histograms. The statistical analyses were performed using the software package IBM®SPSS Statistics for Windows, version 26.

## Results

**Fig. 2:**
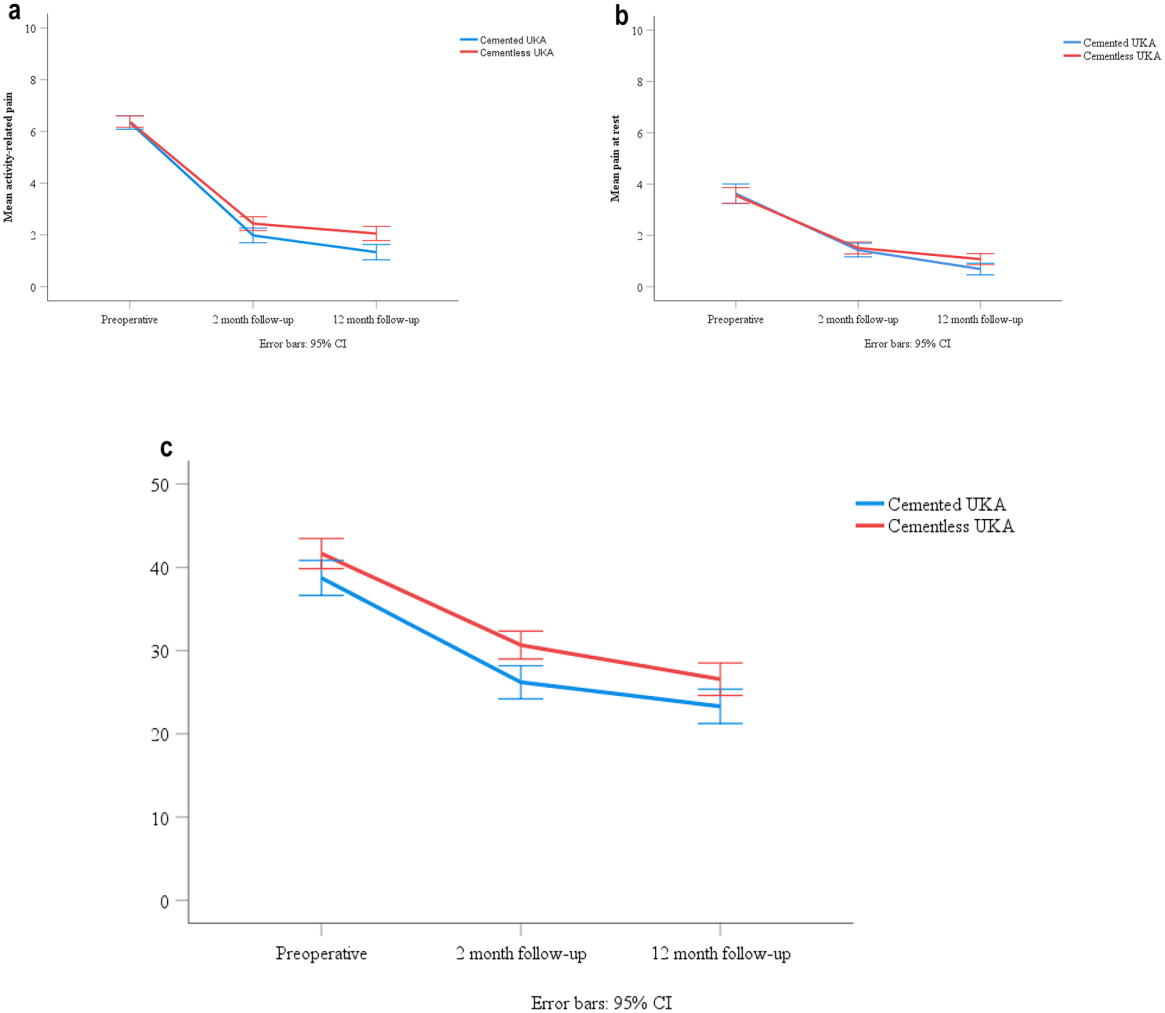
**a** Descriptive activity-related pain score preoperatively and after two- and twelve-months post-operatively. **b** Descriptive pain at rest score preoperatively and after two and twelve-months post-operatively. **c **Descriptive KOOS-PS score preoperatively and after two- and twelve-months post-operatively

The cemented group had significantly less activity-related pain at both follow-up evaluations compared to the cementless group. The descriptive results for activity-related pain, pain at rest and KOOS-PS are presented in Fig. 2a–c. The results of the GLMM model for the various PROMs are presented in Table 2. Subanalyses were performed to address the potential influence of a learning curve with the cementless technique. No clear tendencies were found and all patients were included (results not shown).

**Table 2:** PROMs at two- and twelve-month follow-up, based on a General Linear Mixed Model

	Follow-up	Cemented	Cementless	Difference	*p*-value
Pain, activity	2 month	2.0	2.4	− 0.5	0.024*
Pain, activity	12 month	1.3	2.1	− 0.8	< 0.001*
Pain, rest	2 month	1.4	1.5	− 0.2	0.32
Pain, rest	12 month	0.65	1.1	− 0.4	0.006*
KOOS-PS	2 month	26.9	30.2	− 3.3	0.009*
KOOS-PS	12 month	23.8	26.3	− 2.5	0.072

At the twelve-month follow-up 92% of the patients in the cemented group rated their knee function as better and 91% said that they would have gone through the surgery again. The corresponding numbers in the cementless group were 90% and 88%, respectively.

Mean duration of surgery was 58 min (standard deviation [SD] 10, range 43–100 min) in the cemented group and 54 min (SD 12, range 34–130 min) in the cementless group. Using surgeon as a fixed factor in the GLMM model to account for individual surgeon differences, the estimated duration of surgery in the cemented group was 65 min compared to 57 min in the cementless group (*p* < 0.001).

Distribution of PJIs and other revisions are described in Fig. 1. The PJIs in the cementless group were distributed evenly throughout the study period. Most infections in both groups (ten out of fourteen) were successfully treated with one or two standardized debridement, antibiotics and implant retentions (DAIRs), one patient underwent one-step revision to TKA, and the remaining three patients needed further revisions before conversion to TKA. The patient undergoing one-step revision to TKA had concomitant anterior cruciate ligament reconstruction performed at the primary surgery and had the longest operating time in that group (130 min).

## Discussion

The main finding from the present study was significantly lower scores for activity-related pain in favor of the cemented group at both two- and twelve-month follow-up. Also, pain at rest at twelve-month follow-up and KOOS-PS at two-month follow-up were significantly lower in the cemented group. Duration of surgery was estimated to be eight minutes less with the cementless technique. A relatively high number of PJIs were found for the cementless group.

The differences in scores between the cemented and the cementless group were less than 1 point, and an important question is whether the statistically significant differences found were clinically significant. Bird and Dickson [7] reported that patients at the upper end of the pain scale usually needed a larger decrease before experiencing pain relief. For the NRS, pain from one to three points is considered mild, whereas, moderate pain ranges from four to six points [10]. The patients in the present study had preoperative mean values around six for activity-related pain and four for pain at rest. Already at the two-month follow-up the NRS was around two in both groups. Bandholm et al. [4] used 1.5 points as the minimal clinically important difference (MID) when evaluating knee pain during strength training after TKA, while Rian et al. [19] used a MID of 0.5 points when evaluating postoperative pain after TKA. Based on these considerations, the differences between the groups found in the present study could be clinically relevant.

Nam et al. [16] found no significant difference in pain when comparing cemented and cementless TKA four to six-weeks postoperatively. However, they discussed the possibility of increased pain during the early postoperative phase prior to biologic fixation in the cementless group [16]. Radiostereometric analyses have shown increased migration of cementless tibial components compared with cemented components during the first-three months following the operation, before stabilizing subsequently [11, 17]. Another study reported slightly more pain at six months with cementless tibial fixation in TKA compared with cemented fixation, and no difference one-year postoperatively [6]. The differences in pain in the present study, with lower pain levels in the cemented group, were still present after twelve months. Improvements for all PROMs were found for both groups from preoperative score to the twelve-month follow-up evaluation. Kerens et al. [12] found comparable clinical results between their groups when comparing 60 cases of cemented UKA with 60 cases of cementless UKA.

PJI is one of the most severe complications following arthroplasty surgery. For the cementless group, there were a relatively high number of infections during the first twelve months post-operation compared with the cemented group. Overweight and prolonged duration of surgery have been reported to increase risk of infection [20]. There were no differences in preoperative BMI between the two groups in the present study. However, with mean BMI close to 30 kg/m^2^, a significant number of the patients were overweight (BMI 25–29 kg/m^2^) or suffered from obesity (BMI > 30 kg/m^2^). The duration of surgery was, as expected, significantly lower in the cementless group. A change in operative technique or implant could possibly have led to an increase in rate of infection for a period of time. The infections in the present study were evenly distributed within the two time periods studied and could not easily be explained by a learning curve for the new fixation technique. A theoretical advantage for cemented implants, that they allow local antibiotics around the implant from the cement, could have supported the findings in the present study. However, this has not been possible to detect in large registry-based or case–control studies [20]. One patient in the present study underwent osteosynthesis of a fissure near the tibial component and one was in need of a revision due to subsidence of the tibial plateau, both in the cementless group. A recent review found comparable incidence of periprosthetic tibial fractures in cemented and cementless UKA, but the authors discussed elements of the cementless technique that could increase the risk of fracture [8].

A randomized controlled study design could have secured a more random distribution of patients in the two groups, but no large baseline differences were seen between the two groups (Table 1). The follow-up evaluations were performed by an experienced physiotherapist and not by an orthopedic specialist, which could possibly be considered a limitation of the study. This decision was made for logistical reasons and to secure evaluation of an independent observer. Several studies reporting results after UKA have their origins in the environment of the developers of the implants and there have been concerns around the reproducibility of such results [13]. In a review, Labek et al. [13] found in that the institution that developed the Oxford unicompartmental knee replacement was involved in more than 50% of publications relating to this technique. In the present study, prospectively collected data from a university hospital were reported and three surgeons were involved. This should strengthen the generalizability of the study. Also, the demographic data correspond well to other publications summarized in a recent review [5]. Together with a follow-up rate well above 90%, the presented results should be very representative of this patient group and thus should be very useful to others. The PROMs in the present study, together with the relatively high number of PJIs in the cementless group, were sufficient to question further use of cementless UKA. The department, therefore, has returned to using the cemented technique for all UKAs.

## Conclusion

UKA patients with cemented implants had lower pain scores during activity two and twelve months postoperatively, and at rest one year after surgery, compared with the cementless group. There was a significant reduction in duration of surgery for the cementless group, but a relatively high number of PJIs were found in that same group.
